# Triglyceride–glucose index and hyperhomocysteinemia within a cardiovascular–kidney–metabolic biomarker network: insights from interpretable machine learning and epidemiological modeling

**DOI:** 10.3389/fnut.2026.1898997

**Published:** 2026-08-05

**Authors:** Hongzhou Liu, Ru Wang, Huadan Zhu, Chuan Song, Li Shi, Manka Zhang, Song Dong, Shuangtong Yan

**Affiliations:** 1 Department of Endocrinology, Aerospace Center Hospital, Beijing, China; 2 Department of Endocrinology, Beijing Jishuitan Hospital, Capital Medical University, Beijing, China; 3 Department of Internal Medicine, Jinxiang Grand Hospital, Jining, Shandong, China; 4 Department of Endocrinology, Second Medical Center, Chinese PLA General Hospital & National Clinical Research Center for Geriatric Diseases, Beijing, China

**Keywords:** biomarker network, cardiovascular–kidney–metabolic syndrome, homocysteine, hyperhomocysteinemia, renal function, triglyceride–glucose index

## Abstract

**Background:**

Although the triglyceride–glucose (TyG) index is an established surrogate marker of insulin resistance and metabolic burden, its relationship with hyperhomocysteinemia (HHcy) within the cardiovascular–kidney–metabolic (CKM) framework remains insufficiently understood. This study aimed to investigate the association between TyG and HHcy and to prioritize CKM-related biomarkers using interpretable machine learning and epidemiological modeling.

**Methods:**

This retrospective cross-sectional study included 31,116 adults undergoing health examinations. HHcy was defined as serum homocysteine >15 μmol/L. The relationships among CKM-related biomarkers were characterized using smooth curve fitting, Spearman correlation heatmaps, and network visualization. An extreme gradient boosting model coupled with SHapley Additive exPlanations was used to evaluate feature importance for HHcy identification. Sequential multivariable logistic regression and restricted cubic spline analyses were performed to assess the linear and nonlinear associations between TyG and HHcy. Subgroup and sensitivity analyses were conducted to assess the robustness of the observed associations, including alternative adiposity adjustment, creatinine-based estimated glomerular filtration rate substitution, and restriction of extreme TyG values.

**Results:**

Network analysis showed that TyG clustered predominantly with adiposity and metabolic indices, whereas homocysteine showed closer connectivity with renal-function-related markers. Within the prespecified CKM-related candidate feature set, creatinine ranked highest for HHcy identification, whereas TyG provided limited incremental discriminatory information beyond basic clinical characteristics. In sequential logistic regression, the TyG–HHcy association changed substantially across adjustment models: a crude positive association was attenuated and became inverse after adjustment for CKM-related covariates. In Model 4, which additionally adjusted for log(C-reactive protein + 1) and serum creatinine, each one-unit increase in TyG was associated with lower odds of HHcy (OR = 0.819, 95% CI: 0.765–0.877). Restricted cubic spline analysis indicated a significant nonlinear association (P-nonlinear < 0.001).

**Conclusion:**

TyG appears to be embedded as a metabolic component within a broader CKM biomarker network, whereas renal-function-related markers, particularly creatinine, are more closely linked to the HHcy phenotype. These findings support a CKM network-based interpretation of the TyG–HHcy relationship and highlight the relevance of metabolic–renal interrelationships in cardiometabolic risk assessment.

## 1 Introduction

Hyperhomocysteinemia (HHcy), characterized by elevated circulating homocysteine (Hcy), has been associated with endothelial dysfunction, oxidative stress, vascular injury, and increased cardiometabolic risk (1, 2). Although Hcy has traditionally been regarded as a cardiovascular risk-related biomarker, its regulation is multifactorial and involves metabolic status, renal function, inflammation, nutritional determinants (such as folate and B-vitamin status), medication use, dietary exposures, genetic variants (including MTHFR-related pathways), and demographic characteristics (1, 3, 4). In particular, renal-function-related abnormalities are closely linked to circulating Hcy levels, suggesting that HHcy may reflect not only vascular risk but also broader systemic cardiometabolic and kidney-related alterations (5–7).

The CKM framework provides an integrated perspective for understanding the interconnection among metabolic dysfunction, kidney-related abnormalities, and cardiovascular risk (8–10). Within this framework, metabolic dysregulation and renal-function-related changes may jointly contribute to downstream vascular and biochemical risk phenotypes (9, 11, 12). However, many previous studies have focused on isolated biomarkers or single exposure–outcome associations, which may not fully capture the network-like relationships among metabolic, inflammatory, renal-function-related, and cardiovascular-risk-related biomarkers (8, 13). A CKM biomarker network approach may therefore help clarify how different biological domains are connected with HHcy and identify which components are more closely related to the Hcy phenotype.

The triglyceride–glucose (TyG) index, calculated from fasting triglycerides and fasting plasma glucose, is widely used as a simple surrogate marker of insulin resistance and metabolic burden (14, 15). Higher TyG levels have been associated with obesity, dyslipidemia, hypertension, diabetes, kidney dysfunction, and cardiovascular outcomes (16–18). Nevertheless, the association between TyG and HHcy, and the relative position of these biomarkers within a broader metabolic–renal biomarker network, remain insufficiently understood. This question is particularly relevant in the CKM context, because TyG may represent a metabolic component, whereas Hcy may be more closely associated with renal-function-related alterations (19).

To address this knowledge gap, we conducted a large retrospective health examination study to investigate the association between TyG and HHcy within a CKM-related biomarker framework. We first characterized baseline CKM-related biomarker profiles according to HHcy status and TyG quartiles. We then used exploratory curve fitting, correlation heatmap, and network visualization to examine the position of TyG and Hcy within the CKM biomarker structure. Interpretable machine learning was applied to prioritize HHcy-related biomarkers, while sequential multivariable logistic regression and restricted cubic spline analysis were used to evaluate the linear and nonlinear associations between TyG and HHcy. Finally, subgroup and sensitivity analyses were performed to assess the robustness of the observed TyG–HHcy association across clinically relevant strata and alternative analytical specifications. We hypothesized that TyG would be embedded within the metabolic component of the CKM network, whereas renal-function-related markers would show a closer relationship with the HHcy phenotype.

## 2 Methods

### 2.1 Study design and population

This retrospective observational study used health examination data from the International Medical Center of the Chinese PLA General Hospital in 2025. The source population comprised adults who underwent routine health examinations at the International Medical Center of the Chinese PLA General Hospital in 2025. The database contained demographic information, anthropometric measurements, blood pressure, fasting biochemical parameters, renal-function-related markers, inflammatory markers, and homocysteine measurements. A complete-case analytical strategy was used for the prespecified variables required for the primary analyses. Of 33,964 adults in the source health examination database, 2,848 participants were excluded because of missing values in one or more prespecified variables, including triglycerides, fasting plasma glucose, homocysteine, C-reactive protein, creatinine, uric acid, urea, age, sex, body mass index, waist circumference, systolic blood pressure, or diastolic blood pressure. The final complete-case analytic cohort comprised 31,116 participants (Supplementary Figure S1; Supplementary Table S1). To assess potential selection bias related to the complete-case strategy, we compared participants included in the final analytic cohort with those excluded because of missing prespecified variables. Participants were classified by matching participant IDs between the original health examination dataset and the final analytic dataset, and demographic and cardiometabolic variables were summarized using available non-missing data. No multiple imputation was performed. To evaluate the potential influence of extreme TyG values, we conducted tail-restriction sensitivity analyses limited to the 1st–99th and 2.5th–97.5th percentiles of the TyG distribution. The study was designed to investigate the association between the TyG index and HHcy within a cardiovascular–kidney–metabolic (CKM)-related biomarker framework. This study was conducted in accordance with the Declaration of Helsinki and was approved by the Ethics Committee of the Chinese PLA General Hospital (Approval No. NCRCG-PLAGH-024010). Because this study was based on retrospective de-identified health examination data, the requirement for written informed consent was waived.

### 2.2 Clinical measurements and variable definitions

Demographic information, anthropometric measurements, blood pressure, and fasting laboratory data were extracted from the health examination database. The analyzed variables included age, sex, body mass index (BMI), waist circumference, systolic blood pressure (SBP), diastolic blood pressure (DBP), fasting plasma glucose (FPG), glycated hemoglobin (HbA1c), triglycerides (TG), total cholesterol (TC), low-density lipoprotein cholesterol (LDL-C), high-density lipoprotein cholesterol (HDL-C), alanine aminotransferase (ALT), aspartate aminotransferase (AST), *γ*-glutamyltransferase (GGT), creatinine, uric acid, urea, C-reactive protein (CRP), and Hcy. CRP was log-transformed as log(CRP + 1) to reduce distributional skewness.

Triglyceride and fasting plasma glucose concentrations were recorded in mmol/L in the source health examination database. Before TyG calculation, triglycerides were converted to mg/dL by multiplying by 88.545 and fasting plasma glucose was converted to mg/dL by multiplying by 18.018. The TyG index was calculated as ln[triglycerides (mg/dL) × fasting plasma glucose (mg/dL)/2]. Unit conversion procedures and distributional quality-control checks are presented in Supplementary Table S1. Participants were further categorized into quartiles according to the TyG distribution.

The primary outcome was HHcy, defined as serum Hcy > 15 μmol/L. A sex-specific definition of HHcy, defined as Hcy > 15 μmol/L in men and >12 μmol/L in women, was used in sensitivity analysis. Age was categorized as <60 and ≥60 years. BMI was categorized according to Chinese criteria as normal weight (<24 kg/m^2^), overweight (24–27.9 kg/m^2^), and obesity (≥28 kg/m^2^) (20). Elevated blood pressure was defined as SBP ≥ 140 mmHg or DBP ≥ 90 mmHg. Abnormal glucose metabolism was defined as FPG ≥ 6.1 mmol/L or HbA1c ≥ 5.7%.

### 2.3 Exploratory CKM biomarker network and machine learning analyses

Exploratory analyses were first conducted to characterize the position of TyG within the CKM-related biomarker network. Smooth curve fitting was used to visualize the relationships of TyG with Hcy, log(CRP + 1), creatinine, and uric acid. Spearman correlation matrices were generated for CKM-related biomarkers, including metabolic, anthropometric, blood pressure, liver enzyme, inflammatory, renal-function-related, and Hcy-related markers. Correlation heatmaps and network plots were used to visualize the overall CKM biomarker structure.

Machine-learning analyses were prespecified as exploratory feature-prioritization analyses within the CKM biomarker panel. Their primary purpose was to rank the relative contributions of candidate biomarkers to HHcy identification and to determine whether adding TyG or broader CKM-related biomarker sets materially changed internal discrimination. These analyses were not designed to develop, validate, recommend, or deploy a clinical prediction tool. Internal discrimination and calibration summaries were treated as secondary diagnostic information only. No clinical decision threshold, treatment recommendation, or claim of clinical utility was derived from the machine-learning analyses. The dataset was randomly divided into training and testing sets at a 7:3 ratio using a fixed random seed. Because no external validation cohort was available, model performance was evaluated using an internal train–test split. Categorical variables were converted into dummy variables. The predictors included age, sex, BMI, waist circumference, SBP, DBP, HbA1c, TC, LDL-C, HDL-C, ALT, AST, GGT, creatinine, uric acid, urea, log(CRP + 1), and TyG. An extreme gradient boosting (XGBoost) model was trained using a binary logistic objective and AUC as the evaluation metric. The prespecified hyperparameters were max_depth = 3, eta = 0.03, subsample = 0.80, and colsample_bytree = 0.80 (21). The optimal number of boosting rounds was selected using five-fold cross-validation with early stopping.

For comparison, three logistic regression models were constructed: a basic clinical model including age, sex, BMI, waist circumference, SBP, and DBP; a TyG-enhanced model additionally including TyG; and a CKM-related biomarker model additionally including TyG, log(CRP + 1), creatinine, uric acid, and urea. Model discrimination was assessed in the held-out test set using receiver operating characteristic curves, AUCs, and 95% confidence intervals. Pairwise comparisons of AUCs were performed using paired DeLong tests, with Holm adjustment for multiple comparisons. Calibration was evaluated by comparing observed and predicted probabilities across deciles of predicted risk. The XGBoost model was evaluated using an internal train–test split only and was not externally validated. SHapley Additive exPlanations (SHAP) values were calculated from the XGBoost model to quantify feature importance and visualize the contribution of TyG across creatinine strata (22).

### 2.4 Multivariable modeling and nonlinear analysis

Sequential multivariable logistic regression models were used to examine the association between TyG and HHcy. TyG was analyzed both as a continuous variable and according to quartiles. Model 1 adjusted for age and sex. Model 2 further adjusted for BMI, waist circumference, SBP, and DBP. BMI and waist circumference were retained simultaneously because they capture complementary dimensions of general and central adiposity, respectively; collinearity and alternative adiposity-adjustment analyses were subsequently performed to assess the robustness of this specification. Model 3 further adjusted for TC, LDL-C, HDL-C, ALT, AST, GGT, uric acid, and urea. Model 4 additionally included log(CRP + 1) and creatinine to estimate the association after further accounting for inflammatory and renal-function-related markers. This model was used to explore the extent to which the TyG–HHcy association was altered after adjustment for these CKM-related biomarkers, rather than to infer a causal direct effect. Because TyG, renal-function-related biomarkers, and HHcy were measured contemporaneously, the nested models were interpreted as conditional association models rather than causal adjustment sequences. Depending on the underlying but unobserved causal structure, kidney-related biomarkers could act as confounders, intermediates, or collider-related variables. Therefore, changes in the TyG estimate across models were interpreted as changes in conditional statistical associations and not as estimates of direct, mediated, or protective effects. Odds ratios (ORs) and 95% confidence intervals (CIs) were reported.

Restricted cubic spline analysis with four knots was performed to evaluate potential nonlinearity in the TyG–HHcy association (23). The spline model adjusted for age, sex, body mass index, waist circumference, systolic blood pressure, diastolic blood pressure, total cholesterol, low-density lipoprotein cholesterol, high-density lipoprotein cholesterol, alanine aminotransferase, aspartate aminotransferase, gamma-glutamyl transferase, uric acid, and urea. Overall and nonlinear associations were assessed using analysis of variance from the fitted model. To assess whether the spline pattern was influenced by extreme TyG values, the same spline model was repeated after restricting the TyG distribution to the 1st–99th and 2.5th–97.5th percentiles. The number of participants and HHcy events across TyG deciles was also summarized.

### 2.5 Subgroup, sensitivity, and statistical analyses

Prespecified subgroup analyses were performed according to sex, age group, BMI category, elevated blood pressure status, and abnormal glucose metabolism status. Subgroup-specific covariates were removed from the corresponding models when appropriate. Interaction analyses were performed by adding interaction terms between TyG and subgroup variables.

Sensitivity analyses were conducted to evaluate the robustness of the findings. These included excluding participants with extreme CRP values, excluding participants with extreme creatinine values, excluding participants with abnormal glucose metabolism, and applying a sex-specific definition of HHcy. Extreme C-reactive protein and creatinine values were defined as values above the 99th percentile of their respective distributions.

Variance inflation factors were calculated for Models 2–4 to assess collinearity. To evaluate the influence of simultaneous adjustment for body mass index and waist circumference, alternative models including body mass index alone or waist circumference alone were fitted. In a renal-function sensitivity analysis, serum creatinine was replaced with estimated glomerular filtration rate calculated using the 2021 CKD-EPI creatinine-based equation. Serum creatinine was measured in μmol/L using an enzymatic method on a Beckman Coulter AU5800 automatic clinical chemistry analyzer with reagents from Beijing Baiding Bio-Technology Co., Ltd.; the assay was calibrated using isotope dilution mass spectrometry (IDMS)-traceable calibrators. Serum creatinine was converted to mg/dL by dividing by 88.4 before applying the equation. The 2021 CKD-EPI creatinine-based equation was applied according to age and sex without a race coefficient (24).

Baseline characteristics were summarized according to HHcy status and TyG quartiles. Continuous variables were presented as mean ± standard deviation or median with interquartile range, as appropriate, and categorical variables as frequencies and percentages. Data processing and analysis were performed using R (Version 4.5.3), along with Zstats v1.0.^1^ All statistical tests were two-sided, and *p* < 0.05 was considered statistically significant.

## 3 Results

### 3.1 Baseline characteristics according to HHcy status and TyG quartiles

Of 33,964 adults in the source health examination database, 2,848 were excluded because of missing values in prespecified variables, leaving 31,116 participants in the final complete-case analytic cohort (Supplementary Figure S1; Supplementary Table S1). Included and excluded participants were broadly similar in age, sex, BMI, fasting plasma glucose, HbA1c, homocysteine, and TyG index, with generally small standardized mean differences, although modest differences were observed for some lipid measures. Triglyceride and fasting plasma glucose concentrations were recorded in mmol/L and converted to mg/dL before TyG calculation; the median TyG index was 8.781 (observed range, 7.425–11.062) (Supplementary Table S1). Baseline clinical and biochemical characteristics of the final analytic cohort were summarized according to HHcy status and TyG quartiles (Table 1; Supplementary Table S2). Participants with HHcy showed a more adverse CKM-related biomarker profile compared with those without HHcy, with differences observed in demographic characteristics, adiposity measures, blood pressure, lipid and glucose metabolism, liver enzymes, and renal-function-related markers. In particular, renal-function-related markers, including creatinine, uric acid, and urea, differed between HHcy groups, supporting the relevance of the kidney-related component in the HHcy phenotype. When participants were stratified by TyG quartiles, higher TyG categories were characterized by progressively higher BMI, waist circumference, blood pressure, FPG, HbA1c, TG, liver enzymes, creatinine, uric acid, and urea, whereas HDL-C decreased across TyG quartiles.

**Table 1:** Baseline characteristics of participants according to hyperhomocysteinemia status.

Variables	Overall (*n* = 31,116)	Normal Hcy (*n* = 23,717)	High Hcy (*n* = 7,399)	*p*-value
Age, years	49.73 ± 8.36	49.59 ± 8.29	50.19 ± 8.56	<0.001
Sex, *n* (%)				<0.001
Male	21,435 (68.9)	15,043 (63.4)	6,392 (86.4)	
Female	9,681 (31.1)	8,674 (36.6)	1,007 (13.6)	
BMI, kg/m^2^	25.18 ± 3.21	25.01 ± 3.23	25.74 ± 3.09	<0.001
Waist circumference, cm	88.24 ± 10.32	87.33 ± 10.42	91.16 ± 9.42	<0.001
Weight, kg	72.56 ± 12.58	71.50 ± 12.63	75.95 ± 11.82	<0.001
Height, cm	169.35 ± 7.63	168.71 ± 7.69	171.42 ± 7.04	<0.001
SBP, mmHg	122.53 ± 17.82	121.50 ± 17.85	125.81 ± 17.30	<0.001
DBP, mmHg	81.90 ± 11.32	81.20 ± 11.23	84.14 ± 11.34	<0.001
FPG, mmol/L	5.66 ± 1.20	5.63 ± 1.20	5.77 ± 1.19	<0.001
HbA1c, %	5.83 ± 0.73	5.81 ± 0.73	5.87 ± 0.74	<0.001
TG, mmol/L	1.49 [1.04, 2.17]	1.45 [1.02, 2.13]	1.60 [1.14, 2.31]	<0.001
TC, mmol/L	4.73 ± 0.87	4.73 ± 0.87	4.72 ± 0.89	0.547
LDL-C, mmol/L	3.17 ± 0.83	3.17 ± 0.83	3.15 ± 0.83	0.061
HDL-C, mmol/L	1.26 ± 0.33	1.27 ± 0.34	1.20 ± 0.31	<0.001
ALT, U/L	23.50 ± 14.13	23.10 ± 14.01	24.79 ± 14.42	<0.001
AST, U/L	19.67 ± 7.03	19.49 ± 6.92	20.26 ± 7.33	<0.001
GGT, U/L	27.00 [17.00, 47.00]	26.00 [16.00, 45.00]	32.00 [20.00, 53.00]	<0.001
Creatinine, μmol/L	68.64 ± 13.30	66.86 ± 13.01	74.35 ± 12.60	<0.001
Uric acid, μmol/L	342.00 [282.00, 404.00]	333.00 [274.00, 396.00]	369.00 [312.00, 427.00]	<0.001
Urea, mmol/L	5.15 ± 1.15	5.10 ± 1.14	5.30 ± 1.16	<0.001
CRP, mg/L	0.08 [0.04, 0.16]	0.08 [0.04, 0.16]	0.09 [0.04, 0.17]	<0.001
Hcy, μmol/L	11.25 [8.35, 14.78]	9.86 [7.62, 12.10]	18.26 [16.34, 21.97]	<0.001
TyG index	8.83 ± 0.61	8.80 ± 0.61	8.91 ± 0.59	<0.001
TyG-BMI	221.81 [196.38, 248.20]	219.35 [193.76, 246.17]	228.86 [204.89, 253.38]	<0.001
TyG-WC	783.82 [692.76, 868.35]	772.00 [679.45, 859.68]	816.38 [740.15, 892.03]	<0.001
TyG-WHtR	4.61 [4.13, 5.07]	4.56 [4.08, 5.03]	4.76 [4.32, 5.18]	<0.001
Non-HDL-C, mmol/L	3.47 ± 0.86	3.46 ± 0.86	3.52 ± 0.86	<0.001
TG/HDL-C ratio	1.24 [0.76, 2.04]	1.19 [0.72, 1.97]	1.39 [0.88, 2.22]	<0.001
LDL-C/HDL-C ratio	2.60 [2.00, 3.25]	2.57 [1.97, 3.22]	2.71 [2.11, 3.32]	<0.001
TC/HDL-C ratio	3.86 [3.14, 4.69]	3.81 [3.09, 4.64]	4.04 [3.31, 4.84]	<0.001
Age group, *n* (%)				<0.001
< 60 years	27,415 (88.1)	21,018 (88.6)	6,397 (86.5)	
≥ 60 years	3,701 (11.9)	2,699 (11.4)	1,002 (13.5)	
BMI category, *n* (%)				<0.001
Normal weight	11,245 (36.1)	9,135 (38.5)	2,110 (28.5)	
Overweight	13,803 (44.4)	10,236 (43.2)	3,567 (48.2)	
Obesity	6,068 (19.5)	4,346 (18.3)	1,722 (23.3)	
Elevated blood pressure, *n* (%)				<0.001
No	21,407 (68.8)	16,849 (71.0)	4,558 (61.6)	
Yes	9,709 (31.2)	6,868 (29.0)	2,841 (38.4)	
Abnormal glucose metabolism, *n* (%)				<0.001
No	15,008 (48.2)	11,741 (49.5)	3,267 (44.2)	
Yes	16,108 (51.8)	11,976 (50.5)	4,132 (55.8)	

Continuous variables are presented as mean ± standard deviation or median [interquartile range], and categorical variables are presented as *n* (%). HHcy was defined as serum homocysteine >15 μmol/L. BMI was categorized according to Chinese criteria: normal weight, BMI <24 kg/m^2^; overweight, 24–27.9 kg/m^2^; and obesity, BMI ≥28 kg/m^2^. Elevated blood pressure was defined as SBP ≥140 mmHg or DBP ≥90 mmHg. Abnormal glucose metabolism was defined as FPG ≥6.1 mmol/L or HbA1c ≥ 5.7%. BMI, body mass index; SBP, systolic blood pressure; DBP, diastolic blood pressure; FPG, fasting plasma glucose; HbA1c, glycated hemoglobin; TG, triglycerides; TC, total cholesterol; LDL-C, low-density lipoprotein cholesterol; HDL-C, high-density lipoprotein cholesterol; ALT, alanine aminotransferase; AST, aspartate aminotransferase; GGT, *γ*-glutamyltransferase; CRP, C-reactive protein; Hcy, homocysteine; TyG, triglyceride–glucose index; WC, waist circumference; WHtR, waist-to-height ratio.

### 3.2 Exploratory CKM biomarker patterns associated with TyG

**Figure 1:**
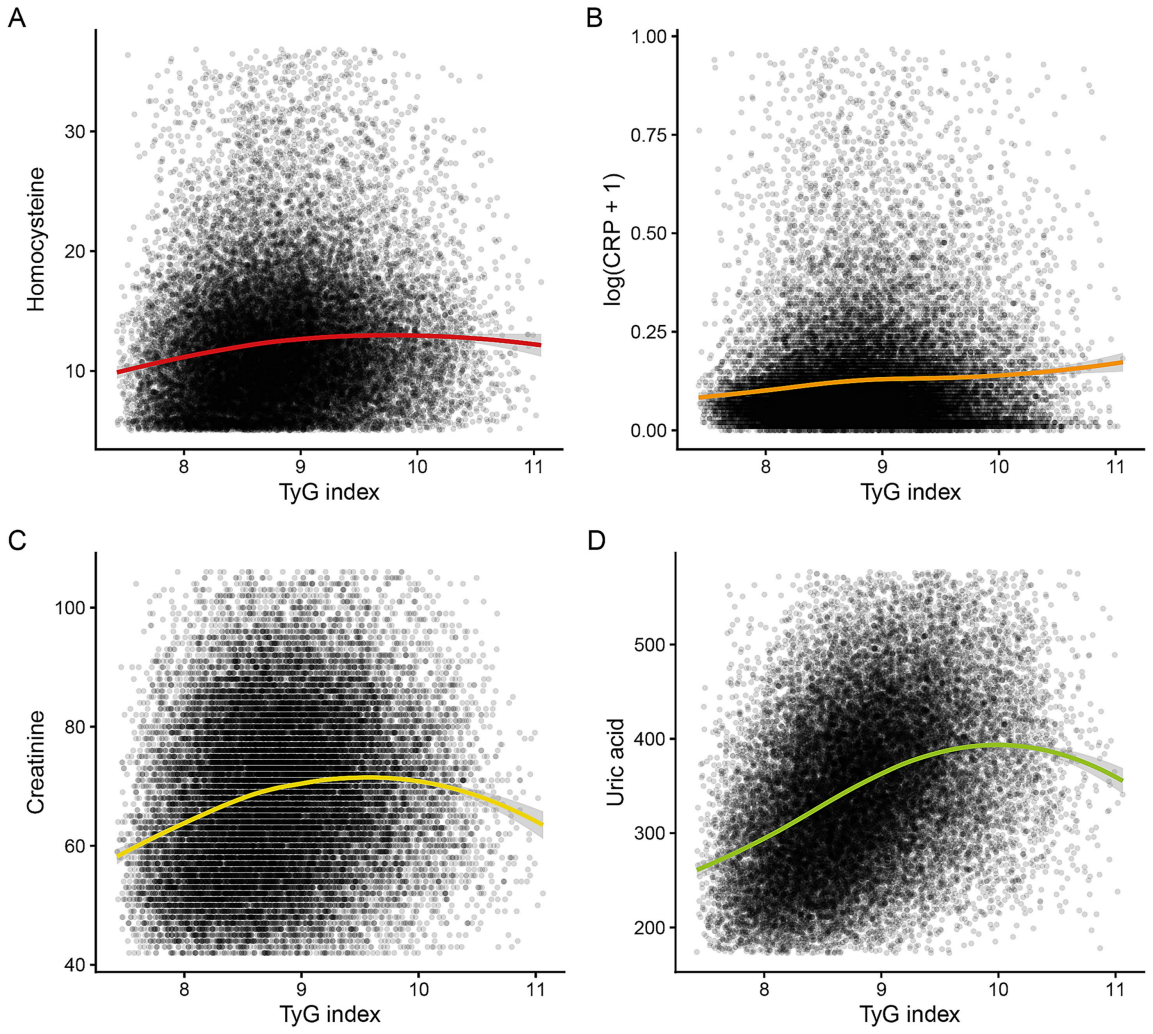
Exploratory associations between TyG and CKM-related biomarkers. Scatter plots with smooth fitted curves showing the exploratory associations between the triglyceride–glucose (TyG) index and selected cardiovascular–kidney–metabolic (CKM)-related biomarkers. **(A)** The association between TyG and homocysteine. **(B)** The association between TyG and log-transformed C-reactive protein [log(CRP + 1)]. **(C)** The association between TyG and creatinine. **(D)** The association between TyG and uric acid. The fitted curves illustrate nonlinear biomarker patterns across the TyG distribution.

Exploratory visualizations were conducted to examine the relationships between TyG and key CKM-related biomarkers. Smooth curve fitting showed nonlinear associations between TyG and homocysteine, log-transformed C-reactive protein [log(CRP + 1)], creatinine, and uric acid (Figure 1). Homocysteine increased modestly across lower-to-middle TyG values and then tended to plateau, whereas creatinine and uric acid showed more evident nonlinear patterns across the TyG distribution.

Spearman correlation heatmap and network analyses further demonstrated substantial interconnectivity among CKM-related biomarkers (Supplementary Figures S2, S3). TyG clustered with metabolic and adiposity-related markers, including triglycerides, body mass index, waist circumference, and TyG-derived indices. Homocysteine showed closer network connectivity with renal-function-related markers, including creatinine, urea, and uric acid.

### 3.3 Exploratory biomarker prioritization within the CKM candidate feature set

Internal model-comparison analyses were conducted only to contextualize biomarker prioritization. Adding TyG to the basic clinical model did not materially improve discrimination (AUC, 0.651 vs. 0.651; ΔAUC = 0.001; Holm-adjusted *p* = 0.705). The broader CKM-related biomarker model showed a statistically significant but modest increase in discrimination compared with the basic clinical model (AUC, 0.679 vs. 0.651; ΔAUC = 0.028; Holm-adjusted *p* < 0.001), whereas XGBoost did not improve discrimination beyond the CKM-related logistic model (AUC, 0.679 vs. 0.679; ΔAUC = 0.001; Holm-adjusted *p* = 0.785) (Supplementary Figure S4; Supplementary Table S3). These internal performance summaries were used to contextualize the relative contribution of biomarker sets and should not be interpreted as external validation, evidence of clinical utility, or support for a deployable HHcy prediction tool.

**Figure 2:**
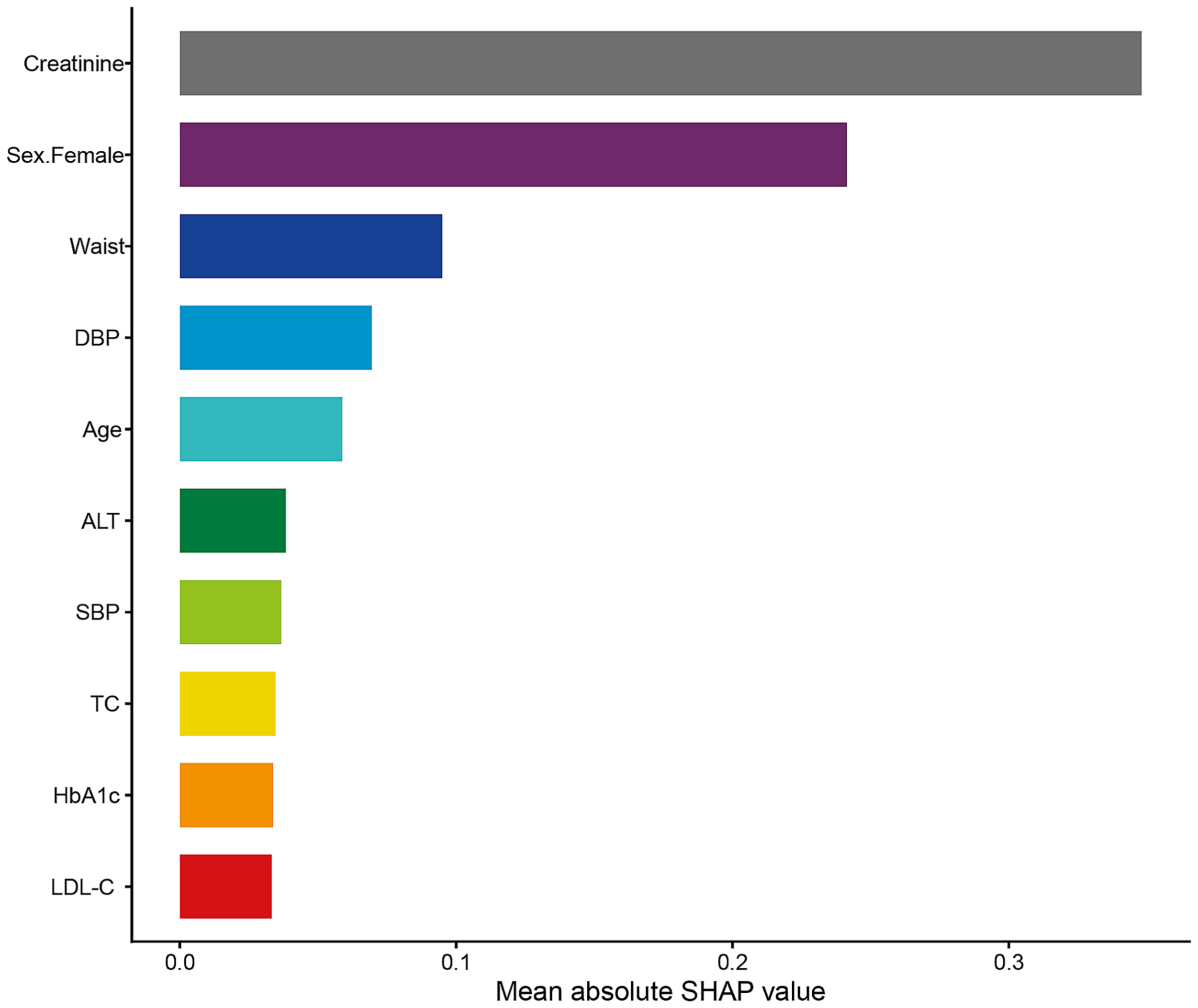
Exploratory SHAP-based prioritization of the top 10 candidate features for HHcy identification. Bar plot showing the 10 candidate features with the largest mean absolute SHAP values in the internally evaluated XGBoost classifier for HHcy identification. Candidate features included demographic, anthropometric, hemodynamic, metabolic, liver-related, and renal-function-related variables. Creatinine ranked highest among the prespecified candidate features, whereas TyG did not rank among the top 10 contributors. SHAP values reflect the relative contribution of each feature to the fitted model output within this dataset and do not establish causal importance, biological primacy, or clinical prediction utility. SHAP, Shapley Additive Explanations; HHcy, hyperhomocysteinemia; TyG, triglyceride–glucose index; XGBoost, extreme gradient boosting.

SHAP-based feature-prioritization analysis ranked creatinine highest for HHcy identification within the evaluated candidate feature set, followed by female sex, waist circumference, diastolic blood pressure, age, alanine aminotransferase, systolic blood pressure, total cholesterol, HbA1c, and LDL-C (Figure 2). TyG did not rank among the top 10 contributors.

The SHAP dependence plot for TyG showed a nonlinear contribution of TyG to model output, with broadly similar patterns across low, middle, and high creatinine strata (Supplementary Figure S5). Overall, SHAP-based feature-prioritization analysis ranked creatinine highest within the evaluated candidate feature set, whereas TyG did not rank among the top 10 contributors. These model-based rankings reflect relative contributions within the fitted internal model and do not establish causal or biological importance.

### 3.4 Multivariable and nonlinear association between TyG and HHcy

**Figure 3:**
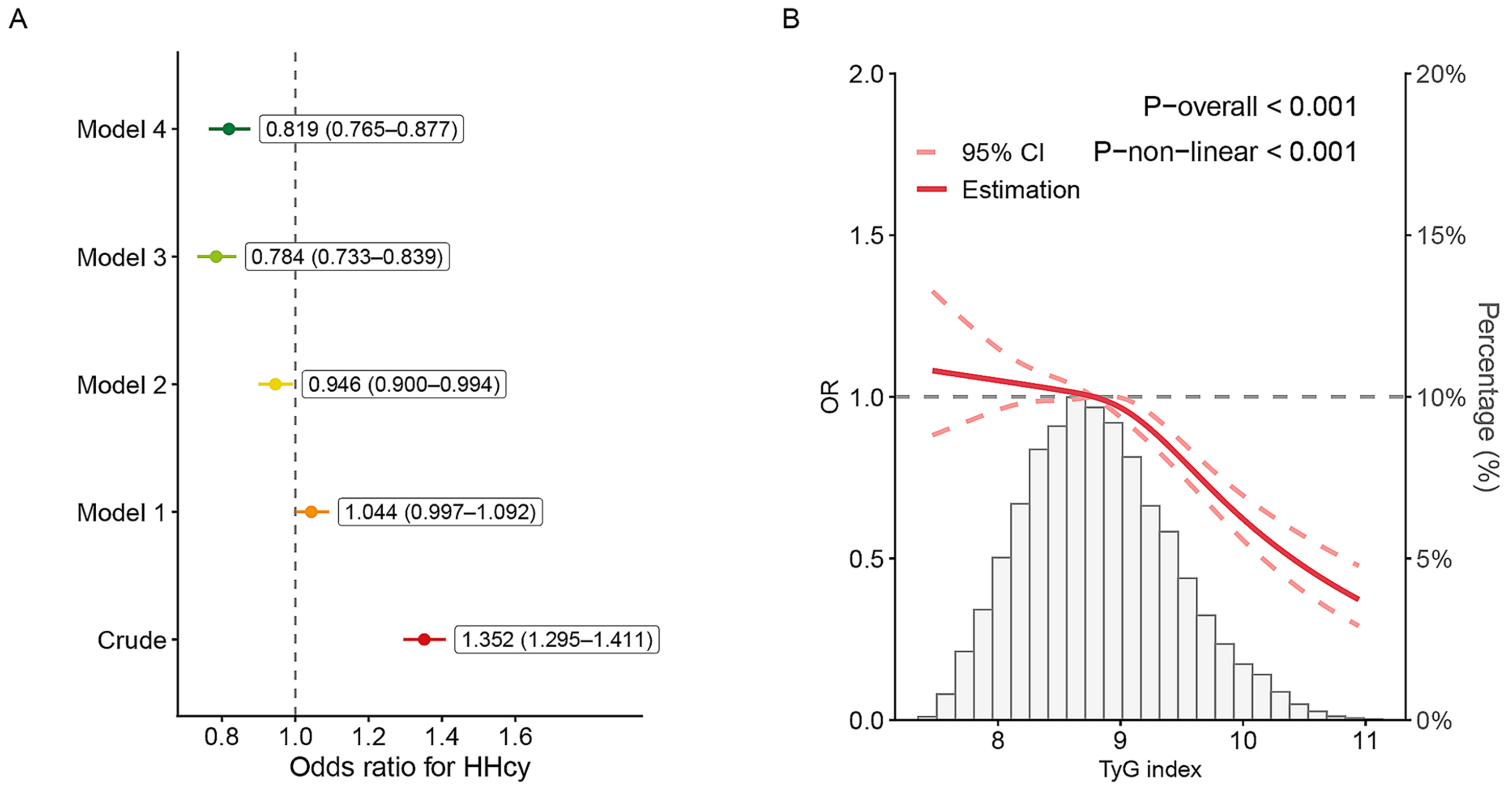
Multivariable and nonlinear associations between TyG and HHcy. Sequential logistic regression and restricted cubic spline analyses of the association between the TyG index and HHcy. **(A)** Odds ratios and 95% confidence intervals for HHcy across sequential adjustment models. The association changed from positive in the crude model to inverse after multivariable adjustment, with Model 4, additionally adjusted for log(C-reactive protein + 1) and serum creatinine, showing an *OR* of 0.819 (95% *CI*: 0.765–0.877). **(B)** The restricted cubic spline curve for the nonlinear association between TyG and HHcy, with a histogram showing the TyG distribution. Odds ratios in the restricted cubic spline analysis were estimated relative to the median TyG index in the analytic cohort. The spline analysis indicated significant overall and nonlinear associations (P-overall < 0.001; P-nonlinear < 0.001). CI, confidence interval; OR, odds ratio.

Sequential logistic regression models showed that the association between TyG and HHcy changed substantially across adjustment models. In the crude model, TyG was positively associated with HHcy. After adjustment for age and sex, this association was markedly attenuated. With further adjustment for adiposity and blood pressure, the association became inverse, and it remained inverse after additional adjustment for lipid profile, liver enzymes, uric acid, and urea. In Model 4, which additionally included log(C-reactive protein + 1) and serum creatinine, each one-unit increase in TyG was associated with lower odds of HHcy (OR = 0.819, 95% CI: 0.765–0.877) (Supplementary Table S4; Figure 3A). Importantly, the direction change occurred before explicit adjustment for serum creatinine. The crude positive association was attenuated after adjustment for age and sex (OR, 1.044) and became inverse after additional adjustment for adiposity and blood pressure in Model 2 (OR, 0.946). The inverse association became stronger after inclusion of lipid-, liver-, uric acid-, and urea-related variables in Model 3 (OR, 0.784), whereas the additional inclusion of serum creatinine in Model 4 modestly attenuated the inverse estimate (OR, 0.819). Thus, the observed direction reversal cannot be attributed solely to creatinine adjustment. Collinearity diagnostics showed VIF values below 3.2 for TyG, body mass index, and waist circumference across sequential models. Moderate collinearity was observed between total cholesterol and LDL cholesterol, as expected from their biological relation. In the extended model, the inverse conditional association between TyG and HHcy remained similar when body mass index and waist circumference were entered separately (Supplementary Table S5).

When TyG was modeled in quartiles, Q2 and Q3 were not clearly associated with HHcy compared with Q1. In Model 3, participants in the highest TyG quartile had lower odds of HHcy than those in the lowest quartile (Q4 vs. Q1: OR = 0.793, 95% CI: 0.714–0.882) (Supplementary Table S4; Supplementary Figure S6). The quartile-based findings were generally consistent with the continuous-variable analysis. In the restricted cubic spline analysis, odds ratios were estimated relative to the median TyG index in the analytic cohort.

Restricted cubic spline analysis confirmed a significant nonlinear association between TyG and HHcy in the full analytic cohort (P-overall <0.001; P-nonlinear <0.001) (Figure 3B). The nonlinear pattern persisted after restricting TyG to the 1st–99th percentile range (P-nonlinear <0.001) and to the 2.5th–97.5th percentile range (P-nonlinear = 0.004) (Supplementary Table S6). TyG deciles contained 3,111–3,112 participants and 505–880 HHcy events per decile, indicating that the observed nonlinear pattern was unlikely to be driven solely by sparse observations at the distributional tails. Given the observed nonlinearity, the per-unit odds ratios from logistic regression should be interpreted as global conditional linear summaries rather than constant effects across the full TyG distribution.

### 3.5 Subgroup and sensitivity analyses

Subgroup analyses showed directionally consistent inverse conditional associations between TyG and HHcy across sex, age group, elevated blood pressure status, and abnormal glucose metabolism status. There was no evidence of interaction by sex, age group, or elevated blood pressure status. Pairwise interaction tests indicated that the TyG–HHcy association differed between overweight and normal-weight participants and between participants with obesity and those with normal weight. Evidence of interaction was also observed by abnormal glucose metabolism status (Supplementary Table S7; Supplementary Figure S7).

Sensitivity analyses yielded similar estimates after excluding participants with extreme C-reactive protein values, excluding participants with extreme creatinine values, excluding participants with abnormal glucose metabolism, and applying a sex-specific HHcy definition (Supplementary Table S7; Supplementary Figure S8). Replacing serum creatinine with creatinine-based estimated glomerular filtration rate produced a similar inverse conditional estimate for TyG (OR, 0.788; 95% CI, 0.736–0.843), compared with the corresponding extended conditional model including serum creatinine (OR, 0.819; 95% CI, 0.765–0.877). The distribution of creatinine-based eGFR in the analytic cohort is also summarized in Supplementary Table S5.

## 4 Discussion

In this large-scale health examination cohort of 31,116 participants, we investigated the relationship between the TyG index and HHcy within a CKM-related biomarker network. Participants with HHcy showed a more adverse CKM-related biomarker profile, particularly with respect to renal-function-related markers. Exploratory network analyses showed that TyG clustered mainly with adiposity and metabolic indices, whereas Hcy showed closer connectivity with renal markers. Within the prespecified CKM-related candidate feature set, creatinine ranked highest for HHcy identification, whereas TyG provided limited incremental discriminatory information. The TyG–HHcy association changed substantially across sequential adjustment models and showed a nonlinear pattern.

The TyG index has been widely used as a surrogate marker of insulin resistance and metabolic burden. In the present study, higher TyG quartiles were accompanied by progressively unfavorable metabolic and anthropometric profiles, including higher BMI, waist circumference, blood pressure, FPG, HbA1c, triglycerides, liver enzymes, creatinine, uric acid, and urea, as well as lower HDL-C. These patterns are consistent with studies showing that TyG and TyG-related indices are associated with metabolic syndrome, obesity-related indices, hypertension, and cardiovascular risk (25–28).

However, our findings indicate that TyG alone did not materially improve HHcy discrimination beyond basic clinical characteristics. Adding TyG increased the AUC by only 0.001 and did not produce a statistically significant improvement after Holm adjustment. Although the CKM-related biomarker model showed statistically higher discrimination than the basic clinical model, the absolute increase in AUC was modest (0.028), and XGBoost did not outperform the CKM-related logistic model. Therefore, the machine learning analyses should be interpreted as internal biomarker prioritization and model comparison rather than evidence for a clinically deployable prediction tool. In the SHAP analysis derived from the XGBoost model, TyG did not rank among the top predictors, while creatinine, sex, waist circumference, blood pressure, age, and several lipid-related or liver enzyme markers showed greater contributions to HHcy identification. These findings suggest that although TyG reflects metabolic burden, its incremental contribution to HHcy identification is modest when considered alongside other CKM-related biomarkers.

This observation is important for interpreting the role of TyG in cardiometabolic research. TyG may represent a metabolic phenotype, whereas HHcy appears to be more closely associated with renal-function-related biomarker alterations. Therefore, a network-based interpretation may be more appropriate than a single-biomarker interpretation when evaluating the TyG–Hcy relationship. Similar context-dependent patterns have been reported in studies examining TyG in relation to CKD, cardiometabolic outcomes, and mortality, especially when obesity, hypertension, diabetes, and other metabolic phenotypes are considered simultaneously (29–32).

Creatinine ranked highest in the SHAP analysis and showed closer network connectivity with Hcy than TyG. This finding should not be interpreted as a novel biological discovery, because the close relationship between renal function and circulating homocysteine is well established (33–35). Rather, the creatinine ranking provides an expected internal benchmark within the present candidate biomarker panel and supports the internal face validity of the feature-prioritization framework.

The contribution of the present analysis is therefore not to establish a new role for creatinine in homocysteine metabolism. Instead, it is to show that, within this health examination cohort and the prespecified CKM-related candidate feature set, renal-function-related information was more informative for HHcy identification than TyG, whereas TyG offered limited incremental discriminatory information beyond basic clinical characteristics. This contrast suggests that TyG should be interpreted as a metabolic-context marker rather than as a leading standalone marker of the HHcy phenotype.

Nevertheless, serum creatinine is not a complete measure of renal function and may be influenced by age, sex, muscle mass, and body composition. In a sensitivity analysis, replacing serum creatinine with 2021 CKD-EPI creatinine-based eGFR yielded a similar inverse conditional TyG estimate, supporting the robustness of the observed conditional association to this alternative creatinine-derived renal-function measure. However, all participants in the analytic cohort had creatinine-based eGFR ≥90 mL/min/1.73 m^2^. Therefore, this analysis should be interpreted as a creatinine-derived renal-function sensitivity analysis rather than as CKD-stage stratification. Moreover, eGFR was derived from creatinine, age, and sex and therefore did not provide an independent kidney biomarker. Albuminuria, cystatin C, and other measures of kidney damage were unavailable; future studies should incorporate these markers to more comprehensively characterize the renal component of the TyG–Hcy relationship (36–38).

A central interpretive issue was the reversal of the TyG–HHcy association across sequential models. In the crude analysis, higher TyG was associated with higher odds of HHcy. This association was substantially attenuated after adjustment for age and sex and became inverse after additional adjustment for adiposity and blood pressure in Model 2. The estimate became more inverse after further inclusion of lipid-, liver-, uric acid-, and urea-related variables in Model 3. By contrast, the subsequent addition of serum creatinine in Model 4 modestly attenuated rather than strengthened the inverse association. Therefore, the direction reversal did not arise solely from adjustment for creatinine.

This pattern should not be interpreted as evidence that higher TyG protects against HHcy. Rather, it may reflect several non-exclusive mechanisms. First, the crude positive association may reflect confounding by correlated CKM risk factors, including adiposity, blood pressure, metabolic abnormalities, and kidney-related biomarker variation. Second, mutual adjustment for highly correlated metabolic measures may produce statistical suppression, such that the residual TyG coefficient represents a conditional contrast different from the unadjusted association. Third, some kidney-related variables may lie on a plausible pathway linking metabolic dysfunction to altered homocysteine handling; adjustment for such variables could remove part of a biologically relevant pathway and introduce overadjustment. Because all biomarkers were measured cross-sectionally, the data cannot distinguish these possibilities or establish a direct, mediated, or protective effect of TyG.

The present findings have several implications. First, HHcy in health examination populations may reflect broader CKM-related biomarker alterations rather than isolated Hcy dysregulation. Participants with HHcy had a more adverse cardiometabolic and renal-function-related profile, suggesting that Hcy should be interpreted within the broader context of CKM risk assessment. This view is consistent with evidence linking Hcy with kidney dysfunction, hypertension-related target organ damage, and cardiovascular risk (35, 39, 40). Second, TyG may be useful for characterizing metabolic burden, but it should not be interpreted as a standalone HHcy predictor. The limited improvement from adding TyG to the basic clinical model, together with its modest SHAP contribution, indicates that TyG is better viewed as one component of a broader biomarker network. This interpretation is consistent with the growing use of interpretable machine learning and SHAP-based approaches for biomarker prioritization rather than simple standalone prediction (41–43). Third, renal-function-related markers should be carefully considered when evaluating the association between metabolic markers and Hcy. Creatinine showed strong relevance across multiple analytic approaches, including machine learning, network analysis, and multivariable regression. These findings suggest that renal-function-related biomarkers may help contextualize the relationship between metabolic dysregulation and Hcy.

From a research perspective, future longitudinal studies are needed to determine whether metabolic abnormalities precede renal-function-related changes and subsequent Hcy elevation, or whether these biomarkers reflect parallel manifestations of shared CKM dysregulation. Incorporating eGFR, albuminuria, cystatin C, dietary factors, folate, vitamin B12, vitamin B6, medication use, genetic determinants, and incident cardiovascular outcomes would further clarify the clinical significance of the observed CKM biomarker network (37, 44, 45). Although lifestyle-related determinants were not measured in the present study, emerging evidence suggests that circadian-aligned dietary patterns, including intermittent fasting and time-restricted eating, as well as physical activity-related metabolic adaptations, may influence broader cardiometabolic and CKM-related phenotypes (46–48). Future longitudinal studies of the TyG–HHcy association should therefore incorporate dietary timing, habitual physical activity, sleep-related behaviors, and other modifiable lifestyle factors to clarify whether they modify or confound the observed biomarker patterns. More broadly, the coexistence of cardiometabolic disease with age-related multisystem conditions, including hearing impairment, underscores the systemic nature of cardiometabolic risk; however, such conditions were not assessed in the present study and should not be interpreted as explanatory factors for the observed TyG–HHcy association (49). Recent evidence also suggests that metabolomic and lifestyle profiles can refine BMI-metabolic phenotypes beyond conventional anthropometric categories, further supporting the need to integrate lifestyle, nutritional, renal, and metabolomic information in future studies of CKM-related biomarker networks (50).

This study has several strengths, including a large health examination cohort, a multidimensional CKM biomarker framework, and the combined use of conventional regression, nonlinear modeling, correlation network visualization, interpretable machine learning, subgroup analysis, and sensitivity analysis. Several limitations should also be acknowledged. First, the retrospective observational design precludes causal inference. TyG, homocysteine, and CKM-related biomarkers were measured at the same health examination visit; therefore, temporal ordering and causal direction could not be established. Second, the study was based on a single-center health examination population, which may limit generalizability to other populations, patients with established cardiovascular disease, chronic kidney disease, or diabetes, and community-based cohorts. Third, although a wide range of CKM-related biomarkers was included, several important determinants of Hcy were unavailable, including folate, vitamin B12, vitamin B6, dietary intake, smoking, alcohol consumption, medication use, genetic polymorphisms, and albuminuria. Fourth, serum creatinine was the primary renal-function-related marker. Although a sensitivity analysis using creatinine-based eGFR yielded similar results, eGFR was derived from creatinine, age, and sex and did not substitute for direct measures of kidney damage. In addition, all participants had creatinine-based eGFR ≥90 mL/min/1.73 m^2^, precluding CKD-stage stratification. Data on urinary albumin-to-creatinine ratio, cystatin C, and cystatin C-based kidney function assessment were unavailable. Fifth, the machine learning models were used for internal biomarker prioritization and performance comparison rather than for developing a deployable clinical prediction tool; no external validation cohort was available. Finally, although included and excluded participants were broadly similar in most demographic and cardiometabolic variables, modest differences in some lipid measures were observed; therefore, selection bias related to the complete-case strategy cannot be completely excluded. Residual confounding, multicollinearity, overadjustment, and collider bias also remain possible.

## 5 Conclusion

In this large health examination cohort, TyG was embedded within a metabolic cluster of the CKM biomarker network, whereas Hcy showed closer connectivity with renal-function-related markers. Within the prespecified CKM-related candidate feature set, creatinine ranked highest for HHcy identification. The TyG–HHcy relationship was nonlinear and changed substantially after adjustment for CKM-related covariates, indicating that TyG should be interpreted as a metabolic component within a broader CKM biomarker network rather than as a standalone determinant of HHcy. Within the evaluated CKM-related biomarker set, renal-function-related information was more informative for HHcy identification than TyG alone. These cross-sectional findings do not establish biological pathways but support longitudinal studies designed to clarify the temporal relationships among metabolic dysfunction, kidney-related alterations, and homocysteine elevation.

## Data Availability

The original contributions presented in the study are included in the article/Supplementary material, further inquiries can be directed to the corresponding author.

## Footnotes

1
http://www.zstats.net
