# Supplementary material for: Triglyceride–glucose index and hyperhomocysteinemia within a cardiovascular–kidney–metabolic biomarker network: insights from interpretable machine learning and epidemiological modeling
Source: Front Nutr. 2026 Aug 5;13:1898997. doi: 10.3389/fnut.2026.1898997 (PMC13485962; doi:10.3389/fnut.2026.1898997)
Supplement: Supplementary file 1 [file Supplementary_file_1.DOCX]

**Supplementary Material**

**Triglyceride–glucose index and hyperhomocysteinemia within a cardiovascular–kidney–metabolic biomarker network: insights from interpretable machine learning and epidemiological modeling**

**Hongzhou Liu^1,†^, Ru Wang^2,†^, Huadan Zhu^3^, Chuan Song^1^, Li Shi^1^, Manka Zhang^1^, Song Dong^1*^, Shuangtong Yan^4^***

^1^ Department of Endocrinology, Aerospace Center Hospital, No. 15 Yuquan Road, Haidian District, Beijing 100049, China.

^2^ Department of Endocrinology, Beijing Jishuitan Hospital, Capital Medical University, No. 31 Xinjiekou Dongjie, Xicheng District, Beijing 100096, China.

^3^ Department of Internal Medicine, Jinxiang Grand Hospital, No. 17 Jinmanke Avenue, Jining City, Shandong Province 272200, China.

^4^ Department of Endocrinology, Second Medical Center, Chinese PLA General Hospital & National Clinical Research Center for Geriatric Diseases, No. 28 Fuxing Road, Haidian District, Beijing 100853, China.

^†^ Hongzhou Liu and Ru Wang contributed equally to this work and share first authorship.

***Correspondence:**

Song Dong Email: qljs2015@163.com Department of Endocrinology, Aerospace Center Hospital, No. 15 Yuquan Road, Haidian District, Beijing 100049, China.

Shuangtong Yan Email: yanshuangtong@301hospital.com.cn Department of Endocrinology, Second Medical Center, Chinese PLA General Hospital & National Clinical Research Center for Geriatric Diseases, No. 28 Fuxing Road, Haidian District, Beijing 100853, China.

**Supplementary Tables**

**Supplementary Table S1. Cohort selection, data completeness, quality-control checks for TyG calculation, and comparison of included and excluded participants**

**Panel A. Cohort selection**

| **Cohort selection step** | **N** |
| --- | --- |
| Original health examination database | 33,964 |
| Excluded because of missing values in ≥1 prespecified variable required for the primary analyses | 2,848 |
| Final complete-case analytic cohort | 31,116 |

**Panel B. Completeness of required variables within the final complete-case analytic cohort**

| **Variable** | **N in final analytic cohort** | **Missing, n** | **Completeness, %** |
| --- | --- | --- | --- |
| TyG | 31116 | 0 | 100 |
| Homocysteine | 31116 | 0 | 100 |
| CRP | 31116 | 0 | 100 |
| Serum creatinine | 31116 | 0 | 100 |
| Uric acid | 31116 | 0 | 100 |
| Urea | 31116 | 0 | 100 |
| Age | 31116 | 0 | 100 |
| Sex | 31116 | 0 | 100 |
| BMI | 31116 | 0 | 100 |
| Waist circumference | 31116 | 0 | 100 |
| SBP | 31116 | 0 | 100 |
| DBP | 31116 | 0 | 100 |

**Panel C. Unit conversion and quality-control checks for TyG calculation**

| **Component** | **Source unit** | **Conversion/calculation** | **Median** | **Observed range** |
| --- | --- | --- | --- | --- |
| Triglycerides | mmol/L | Converted to mg/dL by multiplying by 88.545 | 1.49 | 0.49–6.77 |
| Fasting plasma glucose | mmol/L | Converted to mg/dL by multiplying by 18.018 | 5.34 | 4.20–12.20 |
| TyG index | — | ln[TG (mg/dL) × FPG (mg/dL) / 2] | 8.781 | 7.425–11.062 |

**Panel D. Comparison of included and excluded participants**

| Characteristics | Overall (n=33964) | Included in complete-case analytic cohort (n=31116) | Excluded because of missing prespecified variables (n=2848) | p | SMD |
| --- | --- | --- | --- | --- | --- |
| Age | 49.66 ± 8.90 | 49.67 ± 8.91 | 49.57 ± 8.69 | 0.573 | 0.011 |
| Sex (%) |  |  |  |  |  |
| Male | 23401 (68.9) | 21435 (68.9) | 1966 (69.0) | 0.891 | 0.003 |
| Female | 10563 (31.1) | 9681 (31.1) | 882 (31.0) |  |  |
| BMI | 25.22 ± 3.45 | 25.22 ± 3.44 | 25.24 ± 3.56 | 0.87 | 0.004 |
| Waist | 89.49 ± 97.07 | 89.28 ± 86.81 | 94.34 ± 227.13 | 0.057 | 0.029 |
| Weight | 72.71 ± 13.22 | 72.69 ± 13.20 | 73.18 ± 13.70 | 0.176 | 0.036 |
| Height | 169.36 ± 7.90 | 169.34 ± 7.90 | 169.80 ± 8.00 | 0.029 | 0.059 |
| SBP | 123.94 ± 123.67 | 122.75 ± 21.34 | 151.24 ± 594.17 | <0.001 | 0.068 |
| DBP | 82.02 ± 12.49 | 82.01 ± 12.51 | 82.15 ± 11.98 | 0.692 | 0.011 |
| FPG | 5.72 ± 1.48 | 5.72 ± 1.47 | 5.71 ± 1.60 | 0.789 | 0.005 |
| HbA1c | 5.85 ± 0.87 | 5.85 ± 0.87 | 5.87 ± 0.93 | 0.285 | 0.021 |
| TG | 1.49 [1.04, 2.18] | 1.49 [1.04, 2.18] | 1.46 [1.01, 2.16] | 0.077 | 0.012 |
| TC | 4.74 ± 0.95 | 4.74 ± 0.93 | 4.83 ± 1.13 | <0.001 | 0.091 |
| LDL_C | 3.16 ± 0.89 | 3.17 ± 0.88 | 2.97 ± 0.90 | <0.001 | 0.224 |
| HDL_C | 1.26 ± 0.36 | 1.26 ± 0.36 | 1.20 ± 0.37 | <0.001 | 0.16 |
| ALT | 24.43 ± 21.61 | 24.43 ± 21.87 | 24.31 ± 17.69 | 0.794 | 0.006 |
| AST | 20.29 ± 12.17 | 20.30 ± 12.18 | 20.19 ± 12.05 | 0.692 | 0.009 |
| GGT | 27.00 [17.00, 47.00] | 27.00 [17.00, 47.00] | 29.00 [17.00, 51.00] | 0.009 | 0.099 |
| Creatinine | 69.01 ± 16.89 | 69.03 ± 16.40 | 68.78 ± 22.48 | 0.502 | 0.012 |
| Uric acid | 342.00 [281.00, 406.00] | 342.00 [281.00, 405.00] | 344.00 [278.00, 410.00] | 0.672 | 0.011 |
| Urea | 5.17 ± 1.29 | 5.17 ± 1.28 | 5.21 ± 1.47 | 0.126 | 0.031 |
| CRP | 0.08 [0.04, 0.16] | 0.09 [0.04, 0.16] | 0.08 [0.04, 0.16] | 0.248 | 0.001 |
| Hcy | 11.21 [8.26, 14.88] | 11.25 [8.30, 14.88] | 10.97 [8.01, 14.72] | 0.027 | 0.036 |
| TyG | 8.83 ± 0.64 | 8.83 ± 0.64 | 8.79 ± 0.65 | 0.004 | 0.059 |

Completeness was assessed within the final complete-case analytic cohort. Participants with missing values in prespecified variables required for the primary analyses were excluded before model fitting. Triglyceride and fasting plasma glucose concentrations were recorded in mmol/L and converted to mg/dL before calculating the TyG index. TyG, triglyceride, and fasting plasma glucose distributions were inspected as part of the analytical quality-control procedure. For Panel D, participants were classified by matching participant IDs between the original health examination dataset and the final analytic dataset. Values among excluded participants were summarized using available non-missing data from the original dataset because exclusion was based on missingness in one or more prespecified variables.

**Supplementary Table S2. Baseline characteristics of participants according to TyG quartiles**

| **Variables** | **Overall (n = 31,116)** | **Q1 (n = 7,780)** | **Q2 (n = 7,781)** | **Q3 (n = 7,776)** | **Q4 (n = 7,779)** | **P value** |
| --- | --- | --- | --- | --- | --- | --- |
| Age, years | 49.73 ± 8.36 | 48.13 ± 8.87 | 50.36 ± 8.30 | 50.55 ± 8.17 | 49.89 ± 7.86 | <0.001 |
| Sex, n (%) |  |  |  |  |  | <0.001 |
| Male | 21,435 (68.9) | 3,637 (46.7) | 5,099 (65.5) | 5,964 (76.7) | 6,735 (86.6) |  |
| Female | 9,681 (31.1) | 4,143 (53.3) | 2,682 (34.5) | 1,812 (23.3) | 1,044 (13.4) |  |
| BMI, kg/m² | 25.18 ± 3.21 | 23.28 ± 3.01 | 24.87 ± 3.01 | 25.92 ± 2.92 | 26.68 ± 2.85 | <0.001 |
| Waist circumference, cm | 88.24 ± 10.32 | 81.50 ± 9.90 | 87.26 ± 9.68 | 90.70 ± 9.13 | 93.49 ± 8.43 | <0.001 |
| Weight, kg | 72.56 ± 12.58 | 65.06 ± 11.31 | 71.14 ± 11.86 | 75.31 ± 11.63 | 78.71 ± 11.22 | <0.001 |
| Height, cm | 169.35 ± 7.63 | 167.08 ± 7.56 | 168.82 ± 7.73 | 170.12 ± 7.52 | 171.39 ± 7.01 | <0.001 |
| SBP, mmHg | 122.53 ± 17.82 | 115.06 ± 16.87 | 121.17 ± 17.42 | 124.84 ± 17.05 | 129.05 ± 16.88 | <0.001 |
| DBP, mmHg | 81.90 ± 11.32 | 77.41 ± 10.63 | 81.00 ± 10.92 | 83.40 ± 10.99 | 85.79 ± 11.02 | <0.001 |
| FPG, mmol/L | 5.66 ± 1.20 | 5.06 ± 0.49 | 5.35 ± 0.70 | 5.70 ± 0.99 | 6.54 ± 1.66 | <0.001 |
| HbA1c, % | 5.83 ± 0.73 | 5.52 ± 0.40 | 5.68 ± 0.52 | 5.86 ± 0.66 | 6.24 ± 1.00 | <0.001 |
| TG, mmol/L | 1.49 [1.04, 2.17] | 0.83 [0.71, 0.95] | 1.27 [1.15, 1.41] | 1.80 [1.60, 2.03] | 2.91 [2.42, 3.71] | <0.001 |
| TC, mmol/L | 4.73 ± 0.87 | 4.47 ± 0.82 | 4.67 ± 0.83 | 4.83 ± 0.88 | 4.95 ± 0.89 | <0.001 |
| LDL-C, mmol/L | 3.17 ± 0.83 | 2.94 ± 0.76 | 3.22 ± 0.79 | 3.34 ± 0.84 | 3.16 ± 0.87 | <0.001 |
| HDL-C, mmol/L | 1.26 ± 0.33 | 1.50 ± 0.34 | 1.30 ± 0.30 | 1.18 ± 0.26 | 1.04 ± 0.24 | <0.001 |
| ALT, U/L | 23.50 ± 14.13 | 17.57 ± 10.36 | 21.62 ± 12.55 | 25.21 ± 14.13 | 29.60 ± 15.96 | <0.001 |
| AST, U/L | 19.67 ± 7.03 | 17.82 ± 5.71 | 19.01 ± 6.30 | 20.04 ± 6.98 | 21.81 ± 8.24 | <0.001 |
| GGT, U/L | 27.00 [17.00, 47.00] | 16.00 [12.00, 25.00] | 24.00 [16.00, 36.00] | 32.00 [21.00, 51.00] | 45.00 [29.00, 74.00] | <0.001 |
| Creatinine, μmol/L | 68.64 ± 13.30 | 64.48 ± 12.88 | 68.54 ± 13.41 | 70.44 ± 13.11 | 71.10 ± 12.78 | <0.001 |
| Uric acid, μmol/L | 342.00 [282.00, 404.00] | 290.00 [244.00, 348.00] | 330.00 [277.00, 389.00] | 361.00 [305.00, 417.00] | 386.00 [328.00, 441.00] | <0.001 |
| Urea, mmol/L | 5.15 ± 1.15 | 5.02 ± 1.16 | 5.13 ± 1.14 | 5.18 ± 1.13 | 5.26 ± 1.15 | <0.001 |
| CRP, mg/L | 0.08 [0.04, 0.16] | 0.08 [0.04, 0.12] | 0.08 [0.04, 0.16] | 0.09 [0.04, 0.18] | 0.09 [0.04, 0.19] | <0.001 |
| Hcy, μmol/L | 11.25 [8.35, 14.78] | 10.32 [7.52, 13.75] | 11.12 [8.30, 14.63] | 11.66 [8.73, 15.25] | 11.83 [8.93, 15.43] | <0.001 |
| TyG index | 8.83 ± 0.61 | 8.09 ± 0.21 | 8.59 ± 0.11 | 8.99 ± 0.12 | 9.64 ± 0.34 | <0.001 |
| TyG-BMI | 221.81 [196.38, 248.20] | 186.60 [169.02, 205.13] | 212.01 [194.94, 229.94] | 231.55 [214.18, 250.55] | 255.19 [236.63, 276.63] | <0.001 |
| TyG-WC | 783.82 [692.76, 868.35] | 655.30 [592.64, 720.59] | 749.44 [690.05, 808.83] | 817.54 [760.38, 871.74] | 901.64 [840.95, 960.84] | <0.001 |
| TyG-WHtR | 4.61 [4.13, 5.07] | 3.93 [3.59, 4.27] | 4.43 [4.12, 4.75] | 4.80 [4.49, 5.09] | 5.24 [4.91, 5.59] | <0.001 |
| Non-HDL-C, mmol/L | 3.47 ± 0.86 | 2.97 ± 0.76 | 3.37 ± 0.76 | 3.65 ± 0.81 | 3.91 ± 0.83 | <0.001 |
| TG/HDL-C ratio | 1.24 [0.76, 2.04] | 0.56 [0.44, 0.71] | 1.00 [0.81, 1.22] | 1.57 [1.27, 1.92] | 2.90 [2.20, 3.95] | <0.001 |
| LDL-C/HDL-C ratio | 2.60 [2.00, 3.25] | 1.96 [1.55, 2.45] | 2.53 [2.02, 3.07] | 2.89 [2.35, 3.49] | 3.06 [2.49, 3.67] | <0.001 |
| TC/HDL-C ratio | 3.86 [3.14, 4.69] | 2.98 [2.55, 3.49] | 3.63 [3.13, 4.22] | 4.17 [3.59, 4.81] | 4.83 [4.16, 5.59] | <0.001 |
| Age group, n (%) |  |  |  |  |  | <0.001 |
| <60 years | 27,415 (88.1) | 6,970 (89.6) | 6,770 (87.0) | 6,708 (86.3) | 6,967 (89.6) |  |
| ≥60 years | 3,701 (11.9) | 810 (10.4) | 1,011 (13.0) | 1,068 (13.7) | 812 (10.4) |  |
| BMI category, n (%) |  |  |  |  |  | <0.001 |
| Normal weight | 11,245 (36.1) | 4,806 (61.8) | 3,141 (40.4) | 1,989 (25.6) | 1,309 (16.8) |  |
| Overweight | 13,803 (44.4) | 2,413 (31.0) | 3,456 (44.4) | 3,929 (50.5) | 4,005 (51.5) |  |
| Obesity | 6,068 (19.5) | 561 (7.2) | 1,184 (15.2) | 1,858 (23.9) | 2,465 (31.7) |  |
| Elevated blood pressure, n (%) |  |  |  |  |  | <0.001 |
| No | 21,407 (68.8) | 6,403 (82.3) | 5,621 (72.2) | 5,059 (65.1) | 4,324 (55.6) |  |
| Yes | 9,709 (31.2) | 1,377 (17.7) | 2,160 (27.8) | 2,717 (34.9) | 3,455 (44.4) |  |
| Abnormal glucose metabolism, n (%) |  |  |  |  |  | <0.001 |
| No | 15,008 (48.2) | 5,390 (69.3) | 4,155 (53.4) | 3,232 (41.6) | 2,231 (28.7) |  |
| Yes | 16,108 (51.8) | 2,390 (30.7) | 3,626 (46.6) | 4,544 (58.4) | 5,548 (71.3) |  |

**Notes:** Continuous variables are presented as mean ± standard deviation or median [interquartile range], and categorical variables are presented as n (%). TyG quartiles were defined according to the distribution of the TyG index in the study population. BMI was categorized according to Chinese criteria: normal weight, BMI <24 kg/m²; overweight, 24–27.9 kg/m²; and obesity, BMI ≥28 kg/m². Elevated blood pressure was defined as SBP ≥140 mmHg or DBP ≥90 mmHg. Abnormal glucose metabolism was defined as FPG ≥6.1 mmol/L or HbA1c ≥5.7%. P values represent overall comparisons across the four TyG quartiles and were calculated using one-way analysis of variance for normally distributed continuous variables, the Kruskal–Wallis test for skewed continuous variables, and the chi-square test for categorical variables. TyG quartiles were defined using empirical cut points derived from the analytic cohort.

BMI, body mass index; SBP, systolic blood pressure; DBP, diastolic blood pressure; FPG, fasting plasma glucose; HbA1c, glycated hemoglobin; TG, triglycerides; TC, total cholesterol; LDL-C, low-density lipoprotein cholesterol; HDL-C, high-density lipoprotein cholesterol; ALT, alanine aminotransferase; AST, aspartate aminotransferase; GGT, γ-glutamyltransferase; CRP, C-reactive protein; Hcy, homocysteine; TyG, triglyceride–glucose index; WC, waist circumference; WHtR, waist-to-height ratio.

**Supplementary Table S3. Discrimination performance of clinical, TyG-enhanced, CKM-related biomarker, and XGBoost models for hyperhomocysteinemia in the held-out test set**

**Panel A. Discrimination performance**

| **Model** | **AUC** | **95% CI** |
| --- | --- | --- |
| Basic clinical model | 0.651 | 0.638–0.663 |
| TyG-enhanced model | 0.651 | 0.639–0.664 |
| CKM-related biomarker model | 0.679 | 0.666–0.691 |
| XGBoost model | 0.679 | 0.667–0.691 |

**Panel B. Paired DeLong comparisons**

| **Comparison** | **ΔAUC*** | **P value** | **Holm-adjusted P value** |
| --- | --- | --- | --- |
| Basic clinical model vs TyG-enhanced model | 0.0006 | 0.353 | 0.705 |
| Basic clinical model vs CKM-related biomarker model | 0.028 | <0.001 | <0.001 |
| TyG-enhanced model vs CKM-related biomarker model | 0.028 | <0.001 | <0.001 |
| Basic clinical model vs XGBoost model | 0.029 | <0.001 | <0.001 |
| CKM-related biomarker model vs XGBoost model | 0.001 | 0.785 | 0.785 |

*ΔAUC defined as AUC of the second-listed model minus AUC of the first-listed model.

Notes: The basic clinical model included age, sex, body mass index, waist circumference, systolic blood pressure, and diastolic blood pressure. The TyG-enhanced model additionally included TyG. The CKM-related biomarker model additionally included TyG, log(C-reactive protein + 1), serum creatinine, uric acid, and urea. AUCs and 95% confidence intervals were estimated in the held-out test set. Pairwise model comparisons were performed using the paired DeLong test. Holm adjustment was applied for multiple pairwise comparisons. The XGBoost model was internally evaluated using a single train–test split and was not externally validated.

**Supplementary Table S4. Associations of TyG index with hyperhomocysteinemia in multivariable logistic regression models**

**Panel A. TyG as a continuous variable**

| **Exposure** | **Model** | **Adjustments** | **OR** | **95% CI** | **P value** |
| --- | --- | --- | --- | --- | --- |
| TyG, per 1-unit increase | Crude | None | 1.352 | 1.295–1.411 | <0.001 |
| TyG, per 1-unit increase | Model 1 | Age and sex | 1.044 | 0.997–1.092 | 0.068 |
| TyG, per 1-unit increase | Model 2 | Model 1 + BMI, waist, SBP, DBP | 0.946 | 0.900–0.994 | 0.027 |
| TyG, per 1-unit increase | Model 3 | Model 2 + TC, LDL-C, HDL-C, ALT, AST, GGT, uric acid, urea | 0.784 | 0.733–0.839 | <0.001 |
| TyG, per 1-unit increase | Model 4 | Model 3 + log(CRP + 1) and creatinine | 0.819 | 0.765–0.877 | <0.001 |

**Panel B. TyG quartiles**

| **Exposure** | **Model** |  | **OR** | **95% CI** | **P value** |
| --- | --- | --- | --- | --- | --- |
| Q1 | Model 3 (reference) |  | 1 | Reference | — |
| Q2 vs Q1 | Model 3 |  | 0.995 | 0.914–1.083 | 0.906 |
| Q3 vs Q1 | Model 3 |  | 0.973 | 0.888–1.066 | 0.551 |
| Q4 vs Q1 | Model 3 |  | 0.793 | 0.714–0.882 | <0.001 |

**Notes:** Model 1 adjusted for age and sex. Model 2 further adjusted for BMI, waist circumference, SBP, and DBP. Model 3 further adjusted for TC, LDL-C, HDL-C, ALT, AST, GGT, uric acid, and urea. Model 4 further adjusted for log(CRP + 1) and creatinine. For quartile analyses, Q1 was used as the reference group. OR, odds ratio; CI, confidence interval; TyG, triglyceride–glucose index; HHcy, hyperhomocysteinemia.

**Supplementary Table S5. Collinearity diagnostics and robustness analyses for adiposity and creatinine-based renal-function adjustment in the association between TyG and hyperhomocysteinemia**

**Panel A. Collinearity diagnostics**

| **Variable** | **Model 2: adiposity and blood pressure** | **Model 3: extended covariates** | **Model 4: plus CRP and creatinine** |
| --- | --- | --- | --- |
| TyG | 1.23 | 2.31 | 2.32 |
| Body mass index | 2.79 | 2.87 | 2.87 |
| Waist circumference | 3.12 | 3.17 | 3.18 |
| Total cholesterol | — | 6.2 | 6.2 |
| LDL cholesterol | — | 5.14 | 5.15 |
| HDL cholesterol | — | 2 | 2.02 |
| Alanine aminotransferase | — | 3.34 | 3.33 |
| Aspartate aminotransferase | — | 2.94 | 2.93 |
| All other covariates, maximum VIF | 1.95 | 1.97 | 1.98 |

**Panel B. Robustness to alternative adiposity adjustment**

| **Model specification** | **OR for HHcy per 1-unit higher TyG** | **95% CI** | **P value** |
| --- | --- | --- | --- |
| Model 2: BMI + waist circumference | 0.946 | 0.900–0.994 | 0.027 |
| Model 2: BMI only | 0.973 | 0.927–1.022 | 0.277 |
| Model 2: Waist circumference only | 0.934 | 0.889–0.981 | 0.006 |
| Model 3: BMI + waist circumference | 0.784 | 0.733–0.839 | <0.001 |
| Model 3: BMI only | 0.802 | 0.750–0.858 | <0.001 |
| Model 3: Waist circumference only | 0.78 | 0.729–0.835 | <0.001 |

**Panel C. Creatinine-based eGFR sensitivity analysis**

| **Model specification** | **OR for HHcy per 1-unit higher TyG** | **95% CI** | **P value** |
| --- | --- | --- | --- |
| Model 4: plus CRP and creatinine | 0.819 | 0.765–0.877 | <0.001 |
| Sensitivity model: eGFR replacing creatinine | 0.788 | 0.736–0.843 | <0.001 |

**Panel D. Distribution of creatinine-based eGFR in the analytic cohort**

| eGFR distribution index | Value |
| --- | --- |
| N | 31,116 |
| Mean ± SD, mL/min/1.73 m² | 117.99 ± 7.47 |
| Median [IQR], mL/min/1.73 m² | 118.22 [112.78–122.72] |
| Observed range, mL/min/1.73 m² | 98.36–138.97 |
| eGFR ≥90 mL/min/1.73 m² | 31,116 (100.0%) |
| eGFR 60–89 mL/min/1.73 m² | 0 (0.0%) |
| eGFR <60 mL/min/1.73 m² | 0 (0.0%) |

**Notes:** Model 2 was adjusted for age, sex, body mass index, waist circumference, systolic blood pressure, and diastolic blood pressure. Model 3 additionally included total cholesterol, low-density lipoprotein cholesterol, high-density lipoprotein cholesterol, alanine aminotransferase, aspartate aminotransferase, gamma-glutamyl transferase, uric acid, and urea. Model 4 additionally included log-transformed C-reactive protein and serum creatinine. Moderate collinearity was observed between total cholesterol and LDL cholesterol, as expected from their biological and mathematical relation. VIF values for TyG, body mass index, and waist circumference were all below 3.2. In the renal-function sensitivity analysis, serum creatinine was measured in μmol/L using an enzymatic method on a Beckman Coulter AU5800 automatic clinical chemistry analyzer with reagents from Beijing Baiding Bio-Technology Co., Ltd.; the assay was calibrated using IDMS-traceable calibrators. Serum creatinine was converted to mg/dL by dividing by 88.4 before applying the 2021 CKD-EPI creatinine-based equation without a race coefficient. All participants in the analytic cohort had creatinine-based eGFR ≥90 mL/min/1.73 m²; therefore, the eGFR analysis should be interpreted as a creatinine-derived renal-function sensitivity analysis rather than as CKD-stage stratification. ORs represent the association with hyperhomocysteinemia per one-unit higher TyG index.

**Supplementary Table S6. Robustness of the nonlinear association between the triglyceride–glucose index and hyperhomocysteinemia after restriction of extreme TyG values**

**Panel A. Restricted cubic spline robustness after tail restriction**

| **Analytic sample** | **N** | **Observed TyG range** | **P for overall association** | **P for nonlinearity** |
| --- | --- | --- | --- | --- |
| Full TyG range | 31,116 | 7.425–11.062 | <0.001 | <0.001 |
| Restricted to TyG P1–P99 | 30,492 | 7.667–10.368 | <0.001 | <0.001 |
| Restricted to TyG P2.5–P97.5 | 29,561 | 7.775–10.150 | <0.001 | 0.004 |

**Panel B. Distribution of TyG values and HHcy events across deciles**

| **TyG decile** | **Observed TyG range** | **N** | **HHcy events, n (%)** |
| --- | --- | --- | --- |
| D1 | 7.425–8.069 | 3,112 | 505 (16.2) |
| D2 | 8.069–8.294 | 3,112 | 583 (18.7) |
| D3 | 8.294–8.471 | 3,112 | 670 (21.5) |
| D4 | 8.471–8.627 | 3,112 | 747 (24.0) |
| D5 | 8.627–8.781 | 3,112 | 703 (22.6) |
| D6 | 8.781–8.942 | 3,112 | 793 (25.5) |
| D7 | 8.942–9.115 | 3,111 | 840 (27.0) |
| D8 | 9.115–9.337 | 3,111 | 839 (27.0) |
| D9 | 9.337–9.644 | 3,111 | 880 (28.3) |
| D10 | 9.644–11.062 | 3,111 | 839 (27.0) |

**Panel C. Adjusted linear association after restriction of extreme TyG values**

| **Analytic sample** | **N** | **Adjusted OR for HHcy per 1-unit higher TyG** | **95% CI** | **P value** |
| --- | --- | --- | --- | --- |
| Full TyG range | 31,116 | 0.784 | 0.733–0.839 | <0.001 |
| Restricted to TyG P1–P99 | 30,492 | 0.784 | 0.731–0.841 | <0.001 |
| Restricted to TyG P2.5–P97.5 | 29,561 | 0.796 | 0.739–0.856 | <0.001 |

**Notes:** Restricted cubic spline models used four knots and were adjusted for age, sex, body mass index, waist circumference, systolic blood pressure, diastolic blood pressure, total cholesterol, low-density lipoprotein cholesterol, high-density lipoprotein cholesterol, alanine aminotransferase, aspartate aminotransferase, gamma-glutamyl transferase, uric acid, and urea. Tail-restriction analyses excluded participants outside the specified TyG percentiles while retaining the same outcome definition and covariate adjustment set. Panel B presents unadjusted descriptive event distributions. Given evidence of nonlinearity, the odds ratios in Panel C represent global conditional linear summaries and should not be interpreted as constant effects across the full TyG distribution.

**Supplementary Table S7. Subgroup and sensitivity analyses of the association between TyG index and hyperhomocysteinemia**

**Panel A. Subgroup analyses**

| **Subgroup** | **Level** | **N** | **Events** | **OR** | **95% CI** | **P value** | **P for interaction versus reference** |
| --- | --- | --- | --- | --- | --- | --- | --- |
| Sex | Male | 21,435 | 6,392 | 0.793 | 0.736–0.853 | <0.001 | Ref. |
|  | Female | 9,681 | 1,007 | 0.769 | 0.645–0.917 | 0.003 | 0.393 |
| Age group | <60 years | 27,415 | 6,397 | 0.784 | 0.729–0.843 | <0.001 | Ref. |
|  | ≥60 years | 3,701 | 1,002 | 0.805 | 0.665–0.973 | 0.025 | 0.309 |
| BMI category | Normal weight | 11,245 | 2,110 | 0.88 | 0.775–0.998 | 0.047 | Ref. |
|  | Overweight | 13,803 | 3,567 | 0.771 | 0.699–0.851 | <0.001 | 0.019 |
|  | Obesity | 6,068 | 1,722 | 0.726 | 0.630–0.835 | <0.001 | 0.007 |
| Elevated BP | No | 21,407 | 4,558 | 0.833 | 0.765–0.907 | <0.001 | Ref. |
|  | Yes | 9,709 | 2,841 | 0.745 | 0.667–0.832 | <0.001 | 0.182 |
| Abnormal glucose metabolism | No | 15,008 | 3,267 | 0.809 | 0.721–0.908 | <0.001 | Ref. |
|  | Yes | 16,108 | 4,132 | 0.743 | 0.681–0.812 | <0.001 | 0.037 |

**Panel B. Sensitivity analyses**

| **Analysis** | **OR** | **95% CI** | **P value** |
| --- | --- | --- | --- |
| Excluding extreme CRP values | 0.784 | 0.733–0.840 | <0.001 |
| Excluding extreme creatinine values | 0.781 | 0.730–0.836 | <0.001 |
| Excluding participants with abnormal glucose metabolism | 0.809 | 0.721–0.908 | <0.001 |
| Sex-specific HHcy definition | 0.808 | 0.758–0.860 | <0.001 |

**Notes:** Odds ratios represent the association between TyG and hyperhomocysteinemia per one-unit higher TyG index. Subgroup models were adjusted for age, sex, body mass index, waist circumference, systolic blood pressure, diastolic blood pressure, total cholesterol, low-density lipoprotein cholesterol, high-density lipoprotein cholesterol, alanine aminotransferase, aspartate aminotransferase, gamma-glutamyl transferase, uric acid, and urea, with subgroup-defining covariates removed where appropriate. P values in the final column are derived from TyG-by-subgroup interaction terms and compare each indicated category with the stated reference category. For BMI category, the interaction P values represent pairwise comparisons of overweight and obesity with normal weight. Sensitivity analyses used the same main adjustment strategy. Extreme C-reactive protein and creatinine values were defined as values above the 99th percentile of their respective distributions. BP, blood pressure; CI, confidence interval; CRP, C-reactive protein; HHcy, hyperhomocysteinemia; OR, odds ratio; TyG, triglyceride–glucose index.

**Supplementary Figures**

**Supplementary Figure S1. Flowchart of cohort selection, data processing, and variable derivation**

Note: The raw health examination database included 33,964 participants. After excluding 2,848 participants with missing values in one or more prespecified variables required for the primary analyses, 31,116 participants remained in the complete-case analytic cohort. No multiple imputation was performed. Triglyceride and fasting plasma glucose concentrations were converted to mg/dL before calculation of the triglyceride–glucose index. Age group, body mass index category, and TyG quartiles were subsequently derived. Sensitivity analyses evaluated the robustness of the TyG–hyperhomocysteinemia association after restriction to the 1st–99th and 2.5th–97.5th percentiles of the TyG distribution.

**Supplementary Figure S2. Correlation heatmap of CKM-related biomarkers**

Note: Spearman correlation heatmap showing pairwise correlations among cardiovascular–kidney–metabolic (CKM)-related biomarkers, including metabolic, anthropometric, renal-function-related, inflammatory, liver enzyme, and homocysteine-related variables. Blue indicates positive correlations, whereas red indicates negative correlations.

**Supplementary Figure S3. Correlation network of CKM-related biomarkers**

Note: Network visualization of CKM-related biomarkers based on pairwise correlations. Nodes represent biomarkers, and edges represent correlations between biomarkers. The network illustrates the clustering of TyG-related metabolic markers and the connectivity of homocysteine with renal-function-related markers.

**Supplementary Figure S4. Internal discrimination and calibration summaries for models used in exploratory HHcy biomarker prioritization.**

Note: (A) Receiver operating characteristic curves in the held-out test set for the basic clinical model, TyG-enhanced model, CKM-related biomarker model, and XGBoost model. The basic clinical and TyG-enhanced models achieved AUCs of 0.651, whereas the CKM-related biomarker model and XGBoost model achieved AUCs of 0.679. (B) Calibration curves comparing predicted and observed probabilities of hyperhomocysteinemia across the evaluated models in the held-out test set. The dashed diagonal line indicates ideal agreement between predicted and observed probabilities. The basic clinical model included age, sex, body mass index, waist circumference, systolic blood pressure, and diastolic blood pressure. The TyG-enhanced model additionally included the triglyceride–glucose index. The CKM-related biomarker model additionally included TyG, log-transformed C-reactive protein, serum creatinine, uric acid, and urea. These internal performance summaries were included to contextualize exploratory biomarker prioritization and model comparison; they do not represent external validation, clinical utility, or evidence for a deployable prediction tool. AUC, area under the receiver operating characteristic curve; CKM, cardiovascular–kidney–metabolic; HHcy, hyperhomocysteinemia; TyG, triglyceride–glucose index; XGBoost, extreme gradient boosting.

**Supplementary Figure S5. SHAP dependence plot of TyG stratified by creatinine level**

Note: SHAP dependence plot showing the nonlinear contribution of the TyG index to HHcy identification in the XGBoost model, stratified by low, middle, and high creatinine groups. This descriptive visualization does not constitute a formal test of TyG-by-creatinine interaction and should not be interpreted as evidence of effect modification or a causal biological pathway. SHAP, SHapley Additive exPlanations; TyG, triglyceride–glucose index; HHcy, hyperhomocysteinemia.

**Supplementary Figure S6. Association between TyG quartiles and HHcy**

Note: Forest plot showing odds ratios and 95% confidence intervals for HHcy across TyG quartiles in Model 3, using Q1 as the reference group. Model 3 was adjusted for age, sex, body mass index, waist circumference, systolic blood pressure, diastolic blood pressure, total cholesterol, low-density lipoprotein cholesterol, high-density lipoprotein cholesterol, alanine aminotransferase, aspartate aminotransferase, gamma-glutamyl transferase, uric acid, and urea.

**Supplementary Figure S7. Subgroup analyses of the association between TyG and HHcy**

Note: Forest plot showing subgroup-specific odds ratios and 95% confidence intervals for the association between TyG and HHcy across sex, age group, BMI category, elevated blood pressure status, and abnormal glucose metabolism status. TyG, triglyceride–glucose index; HHcy, hyperhomocysteinemia; BMI, body mass index; BP, blood pressure.

**Supplementary Figure S8. Sensitivity analyses of the association between TyG and HHcy**

Note: Forest plot showing sensitivity analyses for the association between TyG and HHcy after excluding participants with extreme CRP values, excluding participants with extreme creatinine values, excluding participants with abnormal glucose metabolism, and applying a sex-specific HHcy definition. TyG, triglyceride–glucose index; HHcy, hyperhomocysteinemia; CRP, C-reactive protein.
